# Calcitriol, the active form of vitamin D, induces cell death and purinergic signaling modulation in cutaneous melanoma cells

**DOI:** 10.1007/s12032-026-03412-5

**Published:** 2026-09-15

**Authors:** Gilnei Bruno da Silva, Rafael Antônio Narzetti, Paula Dallagnol, Bruna Cristina Ozelame, Daiane Manica, Vítor Henrique Mendes Ramos, Aniela Pinto Kempka, Margarete Dulce Bagatini

**Affiliations:** 1 Multicentric Postgraduate Program in Biochemistry and Molecular Biology, State University of Santa Catarina (UDESC), Lages, SC Brazil; 2 Postgraduate Program in Biochemistry, Federal University of Santa Catarina (UFSC), Florianópolis, SC Brazil; 3 Postgraduate Program in Biomedical Sciences, Federal University of Fronteira Sul (UFFS), Chapecó, SC Brazil; 4 Postgraduate Program in Health Sciences, Community University of the Chapecó Region (Unochapecó), Chapecó, SC Brazil

**Keywords:** Cutaneous melanoma, Vitamin D, Calcitriol, Mitochondria, Ectonucleotidase, Immunity

## Abstract

Cutaneous melanoma (CM) is the most aggressive form of skin cancer, characterized by high metastatic potential and resistance to conventional therapies. Evidence suggests that modulation of purinergic signaling, interleukin-6 (IL-6), and NOD-, LRR-, and pyrin domain-containing protein 3 (NLRP3) may represent promising strategies for treating CM. Calcitriol, the active form of vitamin D, has demonstrated antineoplastic potential across different cancer types. In addition, reports have shown that purinergic signaling can be modulated by calcitriol. However, its mechanism of action on the purinergic system in melanoma remains poorly explored. In this study, we investigated the antineoplastic effects of calcitriol on human cutaneous malignant melanoma cell lines, with a focus on the purinergic system. The cells A375 and SK-MEL-28 were treated with calcitriol at 1, 10, and 50 nM for 24 h, and we analyzed cell viability, mitochondrial membrane potential, apoptotic body detection, migration, and ectonucleotidase enzymatic activity. We also analyzed the expression of ectonucleotidases (CD39 and CD73) and IL-6/NLRP3. We found that calcitriol significantly reduced A375 cell viability, altered mitochondrial potential, inhibited cell migration, and induced the formation of apoptotic bodies. These effects were not observed in SK-MEL-28. Moreover, calcitriol modulated the ectonucleotidases activity and downregulated CD39 and CD73 expression. Additionally, the treatments downregulated IL-6 and NLRP3 expression. Thus, our findings demonstrate that calcitriol exerts antineoplastic effects on melanoma cells via mitochondrial dysfunction, apoptosis-related alterations, inhibition of migration, and modulation of purinergic and inflammatory mediators. Therefore, calcitriol is a promising adjuvant for CM therapy.

## Highlights

Calcitriol treatment reduced the cell viability of A375, but not SK-MEL-28.

Calcitriol altered the mitochondrial transmembrane potential of A375 cells.

The treatment with calcitriol modulated the ectonucleotidases’ enzymatic activity.

Calcitriol downregulated the CD39 and CD73 expression on A375 cells.

Calcitriol downregulated the IL-6 and NLRP3 expression on A375 cells.

## Introduction

Cutaneous melanoma (CM) is a malignant neoplasm arising from deleterious mutations in melanocytes [1]. Due to molecular mutations, there is faster cell proliferation and survival, making CM the most lethal and aggressive cancer affecting the skin [2, 3]. The last epidemiological update reported 331,722 cases and 58,667 deaths from CM worldwide as of 2022 [4]. Although some therapeutics are available to treat CM, low response rates and resistance remain obstacles to its effective management to date [5].

Purinergic signaling, proposed in 1972, is an extracellular signaling pathway involved in homeostatic and pathological processes in the human body [6]. It is composed of receptors called P1 and P2, which, when activated or inactivated, elicit different intracellular signals, ranging from cell proliferation to apoptosis [7]. The signaling molecules, including the nucleotides adenosine triphosphate (ATP) and its derivatives adenosine diphosphate (ADP), adenosine monophosphate (AMP), and the nucleoside adenosine (Ado), play either antagonist or agonist roles depending on their type or on their interactions with their respective receptors on the outer cell membrane [8]. The levels of signaling molecules related to the purinergic system are regulated mainly by the ectonucleotidases (ectoenzymes) known as ecto-nucleoside triphosphate diphosphohydrolase (CD39), ecto 5′-nucleotidase (CD73), and adenosine deaminase (ADA) [9].

Studies had already shown that purinergic signaling is involved in different cancer contexts, such as in gastric cancer [10], breast cancer [11], colorectal cancer [12], pituitary neuroendocrine tumor [13], and in cutaneous melanoma [14, 15]. Furthermore, researchers have proposed using compounds from various sources to modulate the purinergic system and enhance the efficacy of antineoplastic pharmacotherapies [16–19]. Therefore, modulation of the purinergic system may be an interesting strategy to enhance the anticancer effects of pharmacological therapies.

In this context, purinergic signaling and the immune system are closely interconnected [20]. During the inflammatory response, cells can release IL-6 and NLRP3 in response to purinoreceptor stimulation [21–24]. When the scenario presented is about malignant neoplasia, scientific reports show that both IL-6 and NLRP3 roles are controversial, and they can be linked to pro-tumoral outcomes, promoting cancer progression and survival [25, 26]. Thus, these findings suggest that modulation of these two molecules, particularly their downregulation, could improve prognosis in patients with CM.

Given the potential to modulate cell signaling pathways to improve CM therapy, previous studies have shown that calcitriol, the active form of vitamin D, is a promising adjuvant. In this context, a recent in vitro study has shown that calcitriol causes apoptosis in melanoma cells [27]. Calcitriol has also been shown to enhance the anticancer effect of classical pharmacotherapy, inhibit cell proliferation and migration, and induce apoptosis in breast cancer cells [28]. Results from animal experiments showed that calcitriol significantly reduced tumor growth in mice with breast cancer [29]. Studies have also shown that calcitriol is an interesting candidate for modulating IL-6 [30, 31].

Although the aforementioned evidence highlights calcitriol’s potential to combat neoplasia through various cell signaling pathways, the mechanisms by which it acts on the purinergic system and CM remain poorly understood. Thus, in this study, we investigated the antineoplastic effect of calcitriol on two cutaneous melanoma cell lines, focusing on the purinergic signaling. We hypothesized that calcitriol has a potent antineoplastic effect on CM cells by modulating the activity and expression of ectonucleotidases.

## Materials and methods

### Chemicals and reagents

All chemicals and reagents used in this study were purchased from Sigma-Aldrich (St. Louis, MO, USA) and Merck (Darmstadt, Germany) as analytical-grade. The plates, flasks, culture medium, and medium supplies were obtained from Gibco™ Thermo Fisher Scientific (Grand Island, NY, USA) and Invitrogen Life Technologies (Carlsbad, CA, USA). The molecular biology supplies were purchased from Invitrogen and Applied Biosystems (Waltham, Massachusetts, USA).

### Cell line acquisition

We purchased both melanoma cell lines A375 and SK-MEL-28 from the Cell Bank of Rio de Janeiro (CBRJ), Brazil. According to CBRJ, both A375 and SK-MEL-28 are adherent cell lines derived from malignant melanoma. While A375 is classified as an epithelial cell line with epithelial morphology, SK-MEL-28 is a melanoma cell line with a polygonal shape [32].

### Cell culture and treatment

In this study, we used the A375 and SK-MEL-28 cell lines as melanoma models. For A375, the cells were cultured in flasks containing Dulbecco’s modified Eagle’s medium (DMEM), supplemented with high glucose (4,500 mg/L), L-glutamine, and antibiotics/antifungal (1% penicillin/streptomycin and amphotericin B). For SK-MEL-28, the cells were grown under conditions similar to those for A375, except that they were supplemented with a low-glucose medium (1,000 mg/mL). The media were also enriched with 10% fetal bovine serum. Cell culture flasks were maintained in a humidified, controlled atmosphere of 5% carbon dioxide (CO_2_) at 37 °C. After reaching around 80% confluence, cells were trypsinized, seeded into 96- or 6-well plates (according to each subsequent experiment), and treated with 1 nM, 10 nM, and 50 nM of 1ɑ, 25-dihydroxyvitamin D3 (calcitriol) (Cat. Nº H-083, Sigma-Aldrich, St. Louis, MO, USA) for 24 h, following the pre-established study [19]. We performed experimental procedures to discard the per se effect of the vehicle (ethanol 4% group). The control group (CT) received only medium treatment.

### Cell viability assessment

#### MTT assay

For this assay, we used the protocol established by Mosmann [33]. Thus, approximately 2 × 10^4^ cells/well were grown in a 96-well plate and treated with various concentrations of calcitriol for 24 h. The supernatant was removed, and 100 µL of 3-(4,5-dimethyl-2-thiazolyl)-2,5-diphenyl 2 H-tetrazolium bromide (MTT) solution (5 mg/mL in PBS) was added, followed by incubation (2 h at 37 °C). Afterward, the supernatant was discarded, and 200 µL of dimethyl sulfoxide (DMSO) was added to dissolve the formazan crystals released by viable cells. Absorbance was measured at 570 nm (Varioskan™ LUX, Thermo Scientific™). The results were expressed as a percentage (%) of cell viability compared to the control.

#### Microscope fluorescence

Based on the protocol proposed by McGahon et al. [34], with adaptations, we also assessed cell viability using fluorescence microscopy. Approximately 2 × 10^4^ cells/well were cultured in a 96-well plate and treated as described in Sect. 2.2. After the treatment time had elapsed, we removed the supernatant and washed the cells twice with PBS. The cells were stained with a solution of acridine orange (AO) (1 µg/mL) for 5 s. To avoid fluorescence contamination, we discarded the remaining solution and washed the cells twice with PBS. We acquired the readings using a fluorescence microscope (Eclipse TS2-FL, Nikon Corporation™, Tokyo, Japan) (480/490 nm), using a magnification of 200×. We also adjusted the images’ brightness and contrast linearly using ImageJ^®^ (Fiji, version 1.53k). All the experimental procedures were performed in triplicate. The results were expressed as a percentage (%) of fluorescence intensity compared to the control.

### Mitochondrial transmembrane potential

The mitochondrial transmembrane potential was assessed using a previously described protocol [35], with adaptations. After the treatments (as shown in Sect. 2.2 and 2.3.2), we removed the supernatant and washed the cells twice with PBS. Following this, we added 100 µL of Hank’s balanced salt solution (HBSS) containing tetramethylrhodamine ethyl ester (TMRE) (20 nM) and incubated for up to 30 min. Then, we washed the cells with PBS again, gently dried them, and captured images under a fluorescence microscope (Eclipse TS2-FL, Nikon Corporation™, Tokyo, Japan) (549/574 nm), at 200× magnification. The images were linearly adjusted for brightness and contrast using ImageJ^®^ (Fiji, version 1.53k). All procedures were performed in triplicate. The results were expressed as the change in mitochondrial transmembrane potential (ΔΨm) relative to the control.

### Detection of apoptotic bodies and cell counting

Complementary to the cell viability assay, we detected apoptotic bodies and counted cell numbers following the protocol published by Silva et al. [36], with adaptations. This technique was used to demonstrate whether calcitriol can change the cell shape. Briefly, we stained the treated melanoma cells with AO (1 µg/mL) for 5 s and washed them twice with PBS. After gently drying, we captured the images using a fluorescence microscope (Eclipse TS2-FL, Nikon Corporation™, Tokyo, Japan) (480/490 nm), with a magnification of 200×. To clarify the apoptotic body morphotype and DNA fragmentation, we converted the images to grayscale (8-bit) and applied auto-adjustments for brightness and contrast (ImageJ^®^, Fiji, version 1.53k). Then we counted apoptotic bodies and cells. For apoptotic bodies, results were expressed as a percentage (%) relative to the control. Red arrows were used to indicate the apoptotic bodies in images. For cell counting, results were expressed as the number of cells compared to the control.

### Migration assessment

We performed a wound-healing assay to verify melanoma cell migration, as described by Justus et al. [37]. Firstly, we grew 1 × 10^6^ cells/well in 6-well plates until they reached 100% confluence, forming a monolayer. Then, we made a scratch using a sterile 200 µL tip and collected an optical microscopy image at 40× magnification (Eclipse TS2-FL, Nikon Corporation™, Tokyo, Japan). Subsequently, we treated the cells as described in Sect. 2.2. After the treatment time elapsed, we removed the supernatant, washed the cells twice with saline, and took a new photo. To assess wound closure, we selected five points along the wound (from top to bottom) before and after the treatments and drew horizontal lines across from side to side to measure their length in micrometers (µm). Wound closure was determined by the difference in line length before and after the treatments. We adjusted the images linearly for brightness and contrast (software Imagej^®^). The results were expressed as a percentage (%) of wound closure compared to the control.

### Enzymatic activity

We performed an enzymatic assay to verify ectonucleotidase activity in melanoma cells following calcitriol treatment. We used an experimental procedure developed by Lunkes et al. [38] and adapted by Pilla et al. [39] to assess ectonucleotidase activity by measuring their hydrolysis of ATP, ADP, and AMP. Briefly, melanoma cells were cultured in 96-well plates at a density of 2 × 10^4^ cells/well and treated as described in Sect. 2.2. Sequentially, 160 µL of a system solution was added to each well, mixed with 20 µL of specific substrates (ATP, ADP, and AMP), and incubated at 37 °C for 70 min. After the reaction time had elapsed, we added 150 µL of 10% trichloroacetic acid (TCA) to each well to stop the hydrolytic reactions. We then transferred 30 µL of the supernatant to a new 96-well plate and added 300 µL of malachite green solution to detect inorganic phosphate generated by nucleotide hydrolysis. Absorbance was measured at 630 nm (Thermo Scientific™ Varioskan™ LUX). The results were expressed as nmol Pi/min/mg of protein.

For the ADA activity, we followed the previously proposed protocol [40] with adaptations. Firstly, we cultured the melanoma cells in the same manner as aforementioned. Then, we mixed with an adenosine (Ado) solution (21 mM, pH 6.5) and incubated at 37 °C for 60 min. Finally, we added a revelation solution (167.8 mM sodium nitroprusside, 106.2 mM phenol, and 5% sodium hypochlorite) and incubated at 37 °C for 30 min. The absorbance was read at 620 nm, and values were expressed as units per milligram of protein (U/mg of protein).

### Analysis of gene expression

We performed RT-qPCR to analyze gene expression. Thus, we cultured around 1 × 10^6^ cells in 6-well plates and proceeded with the treatments (Sect. 2.2). Afterward, we collected the cells and extracted the RNA using TRIzol™ reagent (Invitrogen™). We verified RNA quantity and quality using a spectrophotometer (Thermo Scientific™ Varioskan™ LUX). We then converted the RNA into cDNA using the High-Capacity cDNA Reverse Transcription Kit (Applied Biosystems™).

RT-qPCR reactions for target amplification were performed using 3 µL of cDNA and 7 µL of PowerUp SYBR Green PCR Master Mix 2× (Thermo Scientific™, USA), up to a final volume of 10 µL per reaction. The RT-qPCR was performed with the following thermal cycling conditions: denaturation at 95 °C for 5 min, followed by 45 cycles of denaturation at 95 °C for 15 s and annealing/extension at 60 °C for 60 s. We analyzed amplicon specificity using a melting curve analysis with a thermal ramp from 60 °C to 95 °C, increasing the temperature by 1 °C every 5 s. The relative expression of the genes was calculated using the comparative ΔΔ^CT^ method. GAPDH was used as the housekeeping gene to normalize gene expression. The oligos sequences (5′-3′) used are shown in Table 1.

**Table 1:** Primer sequences of the investigated genes

Gene	NCBI ID	Sequence (F)	Sequence (*R*)
*GAPDH*	2597	CTCCTCACAGTTGCCATGTA	GTTGAGCACAGGGTACTTTATTG
*CD39*	953	GCCCTGGTCTTCAGTGTATTAG	CTGGCATAACCTACCTACTCTTTC
*CD73*	4907	GTGCCTTTGATGAGTCAGGTAG	TTCCTTTCTCTCGTGTCCTTTG
*IL-6*	3569	TCATCCCATAGCCCAGAGCA	CTGGCATTTGTGGTTGGGTC
*NLRP3*	114,548	CCATCGGCAAGACCAAGA	ACAGGCTCAGAATGCTCATC

### Protein dosage

We determined protein concentrations in cell samples using the Bradford protocol [41]. When necessary, protein concentration was adjusted according to each protocol.

### Statistical analysis

GraphPad Prism 9.0 was used to perform all statistical analyses. All the results were expressed as mean ± standard deviation. One-way ANOVA, followed by Dunnett’s post hoc test, was used to assess differences between the control group (CT) and the calcitriol-treated groups. We considered results statistically significant at *P* < 5% (*P  < 0.05*). Statistical significance was defined as *P  < 0.05, **P  < 0.01, ***P  < 0.001, and ****P  < 0.0001.

## Results

### Effects of calcitriol on malignant melanoma cells’ viability and mitochondrial potential

**Fig. 1:**
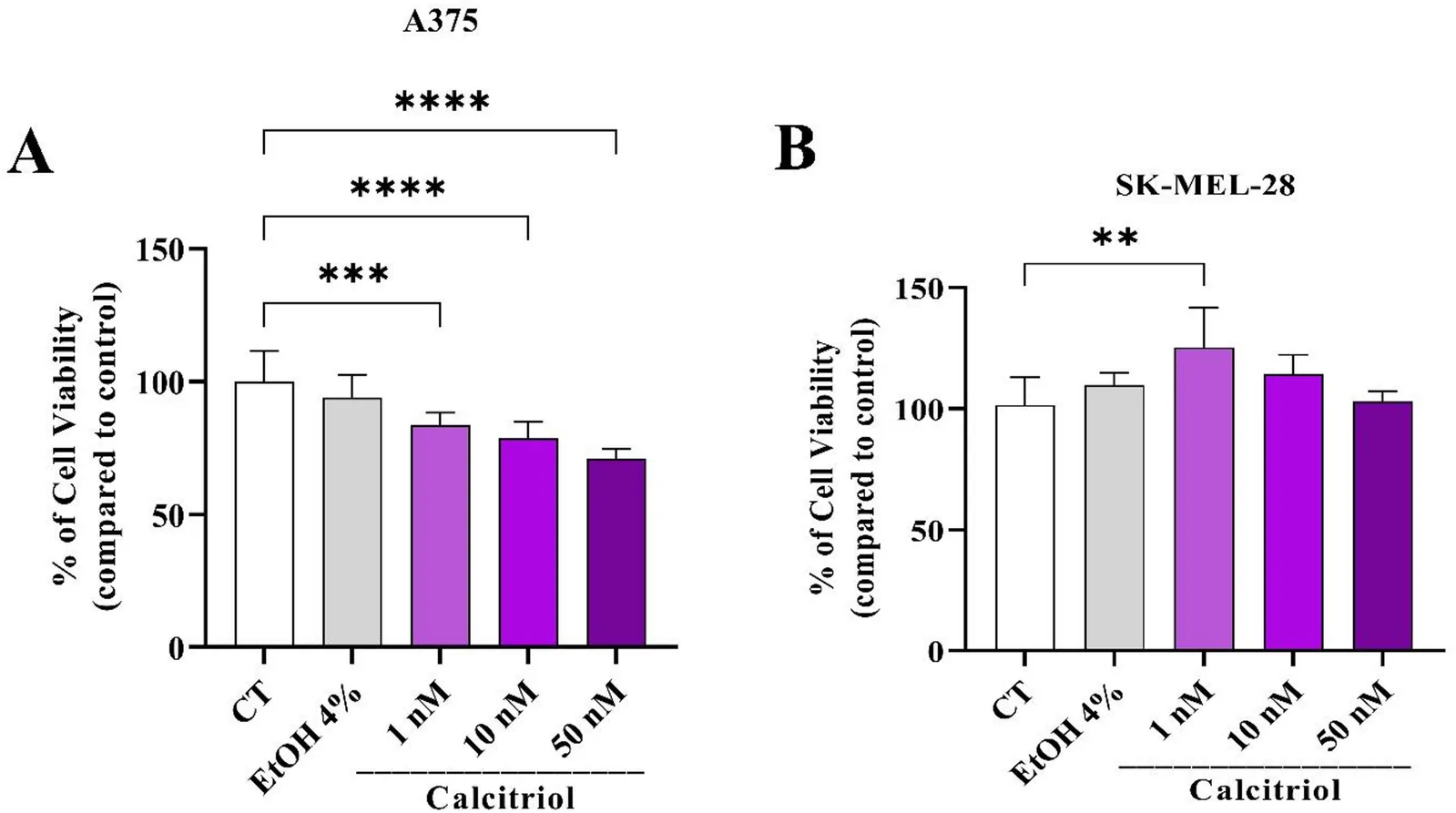
Melanoma cells viability by MTT. Treatment with calcitriol reduced A375 cell viability across all concentrations (**A**). On the other hand, calcitriol did not produce the same effect on SK-MEL-28, but slightly increased cell viability at a concentration of 1 nM (**B**). The experiments were performed at least three times, each with three replicates. Data are presented as mean ± SD. Statistical analysis: one-way ANOVA and post hoc Dunnett’s multiple comparisons test. Representation of statistical significance: **(*P  < 0.01*); ***(P  < 0.001); ****(*P  < 0.0001*)

**Fig. 2:**
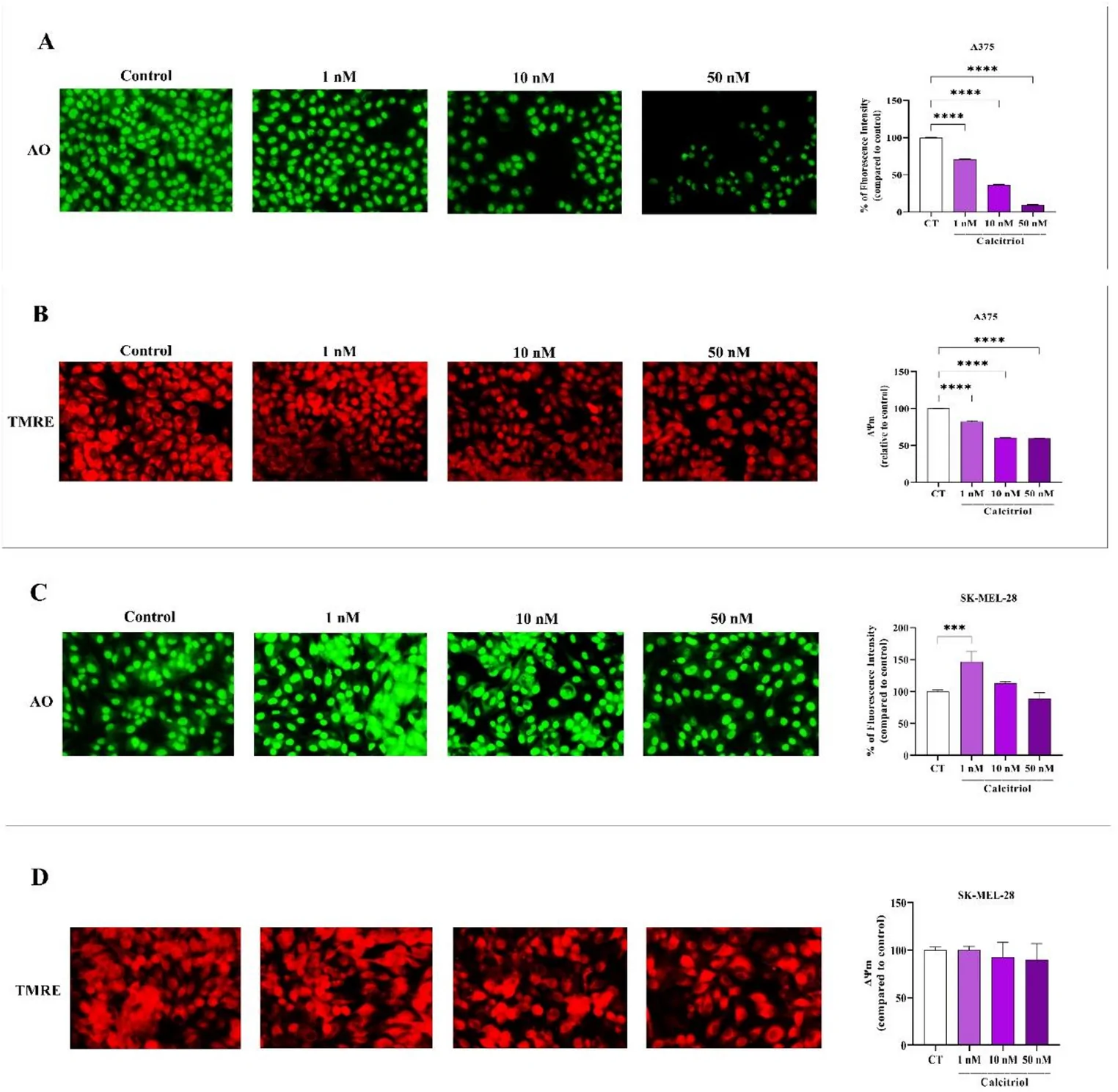
Cell viability by microscope fluorescence and mitochondrial transmembrane potential. A375 and SK-MEL-28 cells were stained with acridine orange (AO) to analyze cell viability and tetramethylrhodamine ethyl ester (TMRE) to measure the mitochondrial transmembrane potential. Calcitriol significantly reduced the A375 cells’ viability at all concentrations tested (**A**) and slightly increased the SK-MEL-28 viability at a concentration of 1 nM (**B**). While all concentrations of calcitriol reduced the mitochondrial transmembrane potential of A375 cells (**C**), it did not present the same effect on the SK-MEL-28 cells (**D**). The experiments were performed at least three times, each with three replicates. Data are presented as mean ± SD. Statistical analysis: one-way ANOVA and post hoc Dunnett’s multiple comparisons test. All images were captured at 200× magnification. Representation of statistical significance: ***(P  < 0.001); ****(*P  < 0.0001*)

We verified that calcitriol had an effect on the viability of the melanoma cells A375 and SK-MEL-28 (Figs. 1 and 2). By the MTT method, we discovered that calcitriol reduced the viability of A375 cells at all concentrations tested when compared to control (1 nM, *P* = 0.0004; 10 nM, *P* < 0.0001; and 50 nM, *P* < 0.0001) (Fig. 1A). However, we did not find the same results for the cells SK-MEL-28, which had an increase in cell viability when it was treated with 1 nM of calcitriol (*P  = 0.0049*) (Fig. 1B). Using fluorescence microscopy for a complementary assessment of cell viability, we corroborated the results found by the MTT, with significantly reducing the fluorescence intensity of A375 cells at all concentrations tested (*P  < 0.0001*) (Fig. 2A). Likewise, the high fluorescence intensity confirmed that 1 nM of calcitriol increased the cell viability of SK-MEL-28 (*P  = 0.0007*) (Fig. 2C).

Converging with the results of cell viability, calcitriol also interfered with the mitochondrial potential (Fig. 2). We found a significant reduction of mitochondrial transmembrane potential for A375 cells at all calcitriol concentrations tested (*P  < 0.0001*) (Fig. 2B). No statistically significant alterations in the mitochondrial potential were observed for SK-MEL-28 cells when treated with calcitriol (Fig. 2D).

### Treatment with calcitriol stimulated apoptotic body formation in A375 cells

**Fig. 3:**
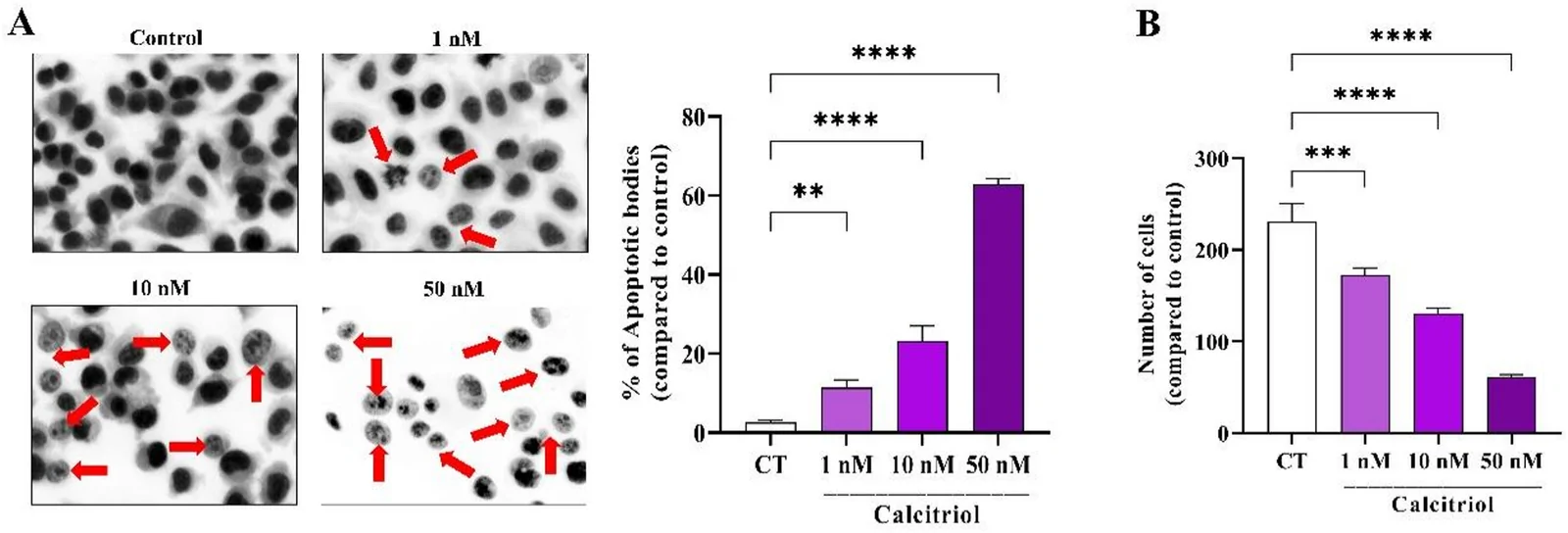
Apoptotic bodies and cell counting of A375 cells. The percentage of apoptotic bodies in A375 cells increased according to calcitriol treatment concentrations (**A**). Conversely, the number of cells counted decreased with increasing calcitriol treatment (**B**). The experiments were performed at least three times, each with three replicates. Data are presented as mean ± SD. Statistical analysis: one-way ANOVA and post hoc Dunnett’s multiple comparisons test. All images were captured at 200× magnification. Representation of statistical significance: **(*P  < 0.01*); ***(P  < 0.001); ****(*P  < 0.0001*)

As shown in Fig. 3, calcitriol treatment for 24 h stimulated nuclear fragmentation and the formation of apoptotic bodies (red arrows). Compared to control, we detected that calcitriol induced apoptotic bodies at concentrations of 1 nM (*P  = 0.0036*), 10 nM (*P  < 0.0001*), and 50 nM (*P  < 0.0001*) (Fig. 3A). Furthermore, the treatment with calcitriol significantly reduced the number of cells at all concentrations (1 nM, *P* = 0.0005; 10 nM, *P* < 0.0001; and 50 nM, *P* < 0.0001) (Fig. 3B).

### A375 cell migration is inhibited by calcitriol

**Fig. 4:**
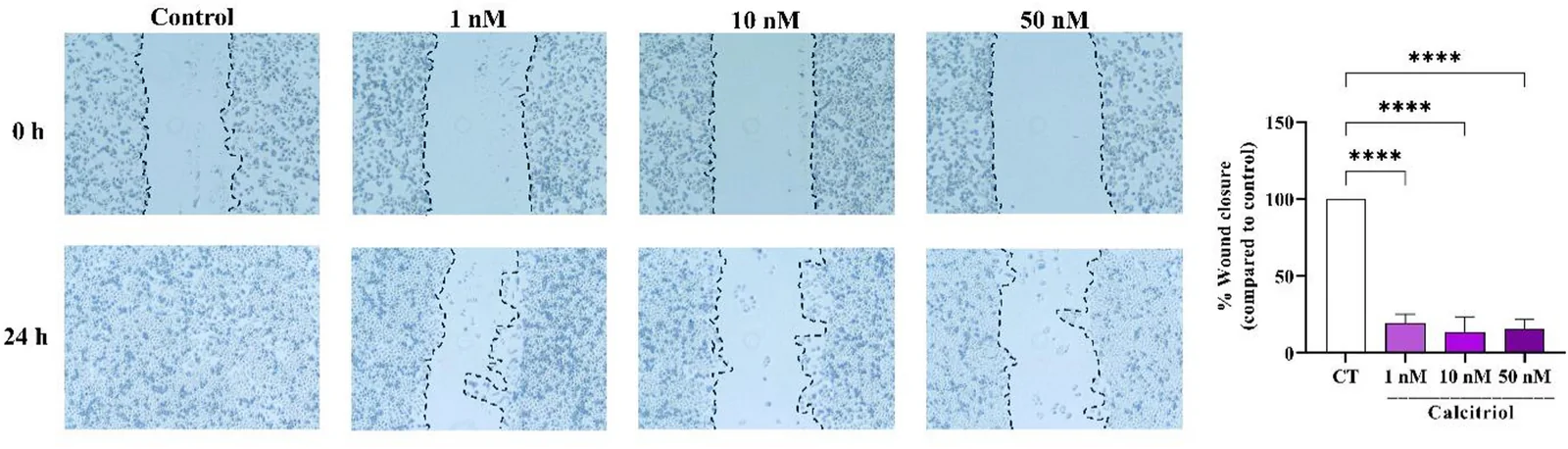
Migration of A375 cells. All tested calcitriol concentrations inhibited migration in A375 cells. The experiments were performed at least three times, each with three replicates. Data are presented as mean ± SD. Statistical analysis: one-way ANOVA and post hoc Dunnett’s multiple comparisons test. All images were captured at 40× magnification. Representation of statistical significance: ****(*P  < 0.0001*)

Figure 4 shows the migration of A375 cells after calcitriol treatment. Notably, calcitriol treatment inhibited cell migration and wound closure across all concentrations tested, with a statistically significant effect (*P  < 0.0001*) at 24 h compared to the control.

### Calcitriol modulated the ectonucleotidase activity in A375 cells

**Fig. 5:**
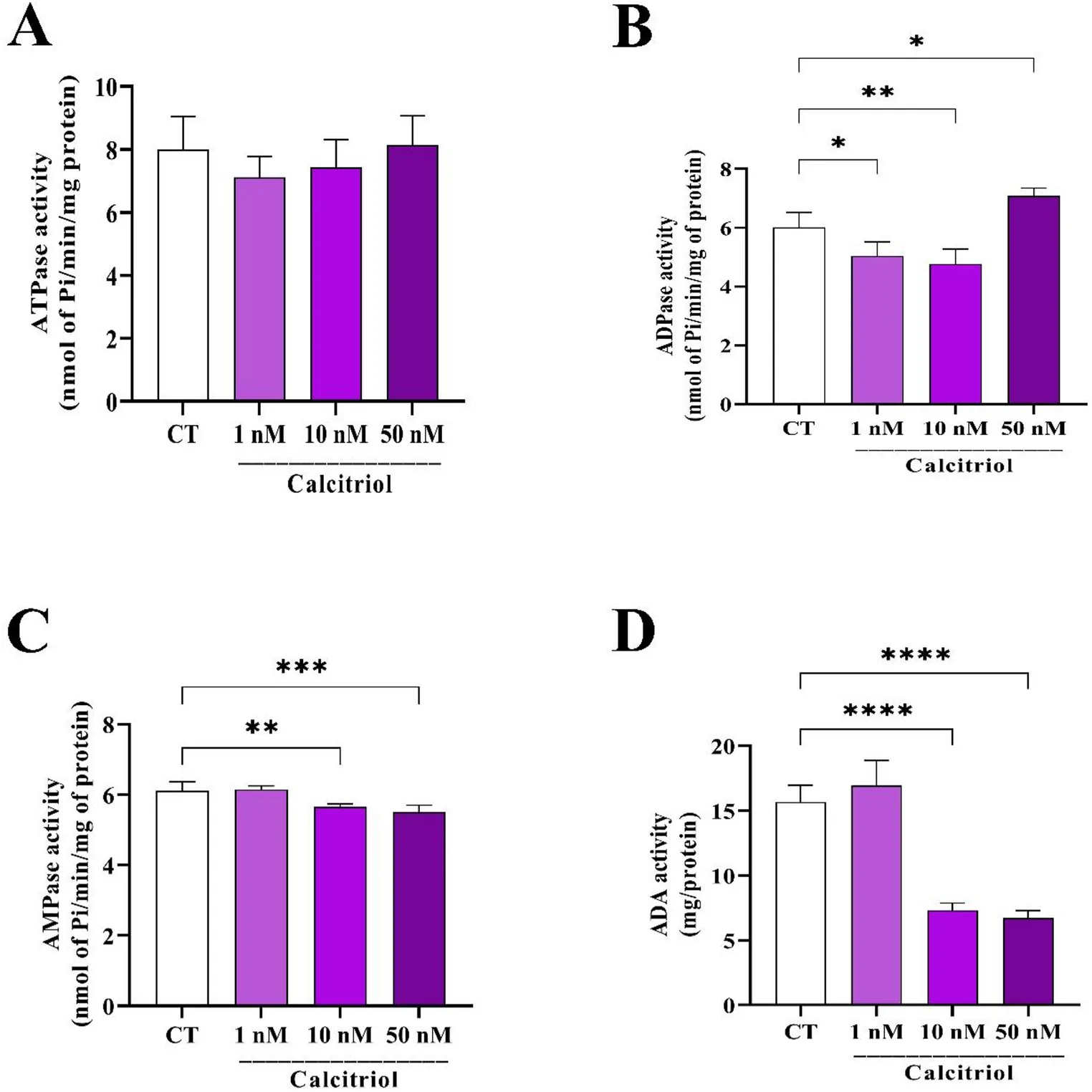
Enzymatic activity on A375 cells. After 24 h, calcitriol treatment did not influence ectonucleotidase activity on ATP hydrolysis (**A**), but decreased ADP hydrolysis at 1 nM and 10 nM and increased it at 50 nM (**B**). Calcitriol treatment decreased the enzymatic activity of ectonucleotidases in the hydrolysis of AMP (**C**) and Ado (**D**). The experiments were performed at least three times, each with three replicates. Data are presented as mean ± SD. Statistical analysis: one-way ANOVA and post hoc Dunnett’s multiple comparisons test. Representation of statistical significance: *(*P  < 0.05*); **(*P  < 0.01*); ***(P  < 0.001); ****(*P  < 0.0001*)

The enzymatic activity of ectonucleotidases after calcitriol treatment is shown in Fig. 5. Calcitriol decreased the hydrolysis of ADP at concentration of 1 nM (*P  = 0.0150*) and 10 nM (*P  = 0.0025*) but increased at concentration of 50 nM (*P  = 0.0204*) when compared to control (Fig. 5B). Conversely, the treatment significantly reduced the hydrolysis of AMP at concentrations of 10 nM (*P  = 0.0055*) and 50 nM (*P  = 0.0007*) (Fig. 5C). The adenosine breakdown also was strongly reduced at concentrations of 10 nM (*P  < 0.0001*) and 50 nM (*P  < 0.0001*) in comparison to control (Fig. 5D). We did not find any statistical significance in the ATP hydrolysis (Fig. 5A).

### Gene expression of CD39, CD73, IL-6 and NLRP3 in A375 cells

**Fig. 6:**
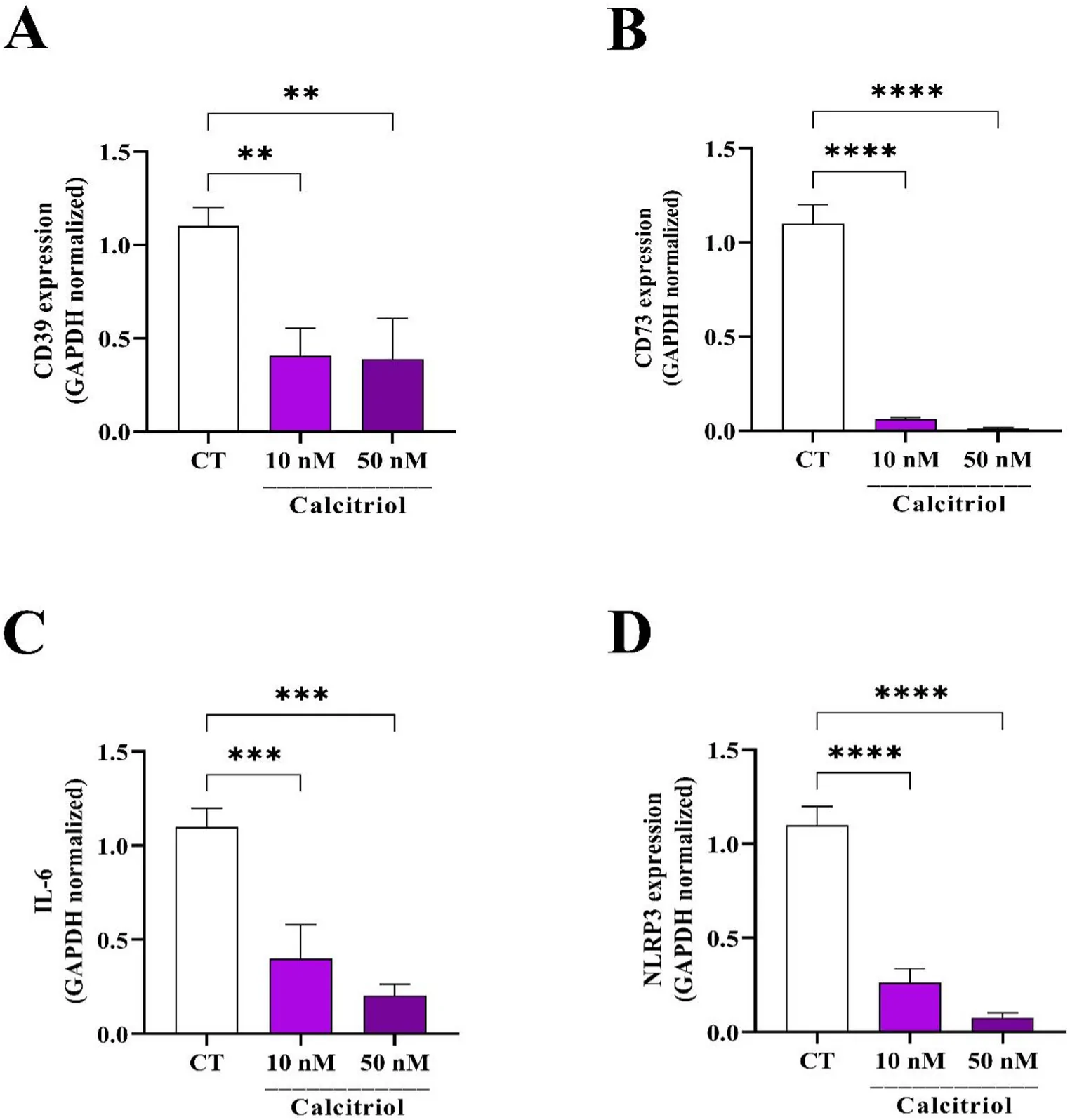
Gene expression. Calcitriol treatment downregulated the expression of CD39 (**A**), CD73 (**B**), IL-6 (**C**), and NLRP3 (**D**) at concentrations of 10 nM and 50 nM. The experiments were performed at least three times, each with three replicates. Data are presented as mean ± SD. Statistical analysis: one-way ANOVA and post hoc Dunnett’s multiple comparisons test. Representation of statistical significance: **(*P  < 0.01*); ***(P  < 0.001); ****(*P  < 0.0001*)

The calcitriol treatment for 24 h was able to modulate the gene expression of the ectonucleotidases and the components of inflammation (Fig. 6). We found that calcitriol downregulated the expression of CD39 at concentrations of 10 nM (*P  = 0.0035*) and 50 nM (*P  = 0.0031*) when compared to control (Fig. 6A). Similarly, the expression of CD73 was also strongly downregulated by calcitriol at concentrations of 10 and 50 nM with the same statistical significance (*P  < 0.0001*) (Fig. 6B). Regarding the elements involved in inflammation, we verified a downregulation of IL-6 at both concentrations analyzed (10 nM, *P* = 0.0008; 50 nM, *P* = 0.0002) compared to control (Fig. 6C). We also verified a significant downregulation of NLRP3 in both concentrations analyzed (10 nM and 50 nM, *P* < 0.0001) in comparison to the control group (Fig. 6D).

## Discussions

A robust body of evidence has shown that vitamin D exhibits properties against various neoplasms, including breast cancer [42], colorectal cancer [43], osteosarcoma [44], cholangiocarcinoma [45], and lung cancer [46]. The active form of vitamin D, calcitriol, has also been shown to have anticancer activity, including in breast cancer [47], prostate cancer [48], and cutaneous melanoma [19, 27]. In this study, we understood the effects of calcitriol against two cutaneous melanoma cell lines, focusing on purinergic signaling. From this, we discovered that calcitriol exhibited antineoplastic effects on cutaneous malignant melanoma cells (A375), altered mitochondrial function, and modulated ectonucleotidase activity and expression.

Using the MTT assay, we first investigated calcitriol’s ability to induce cell death and reduce viability in A375 and SK-MEL-28 cells. Treatment with calcitriol showed distinct effects on cells, decreasing the viability of A375 at all concentrations, but did not affect the viability of SK-MEL-28 (Fig. 1). Using microscope fluorescence, we corroborated the results obtained in MTT, with significant reduction in A375 cell viability at all calcitriol concentrations and no statistical significance in case of SK-MEL-28 (Fig. 2A and C). The patterns observed in this study are very similar to those found by other researchers, such as Domzalski et al., who tested the antiproliferative properties of calcitriol on A375 and SK-MEL-28 cells [49]. However, this antineoplastic effect of calcitriol is not restricted to melanoma and has been demonstrated on colorectal carcinoma [50] and breast cancer cells [51] as well. These results support the potential use of calcitriol for inducing cancer cell death.

The curious result observed in this study, in which calcitriol did not induce death in SK-MEL-28 cells, was also reported by other researchers [19, 49, 52]. A possible explanation for the different role of calcitriol in melanoma cells involves the vitamin D receptor (VDR). Evans et al. showed that SK-MEL-28 has lower functional VDR expression than several other melanoma cell lines, including SK-MEL-2, SK-MEL-5, SK-MEL-24, HT144, and RPMI 7951, thereby rendering SK-MEL-28 resistant to the antiproliferative effects of calcitriol. However, SK-MEL-28 showed sensitivity at a concentration of 10^− 6^ [52]. All these findings explain why, in our study, we did not observe any effect of calcitriol on SK-MEL-28, since the highest concentration we tested was 50 nM.

Recent studies have demonstrated that mitochondria may be a useful target for improving anticancer therapies, particularly to reverse chemoresistance [53–55]. Based on this premise, we searched for alterations in mitochondria and found a significant reduction in the transmembrane potential of this organelle in A375 cells treated with calcitriol, which was not observed in SK-MEL-28 (Fig. 2B and D). The disruption in mitochondrial function observed in this study suggested that the antineoplastic impact of calcitriol may arise from multiple mechanisms of action.

Among the functions of mitochondria in cells, they play an important role in programmed cell death, also known as apoptosis [56]. Apoptosis is orchestrated by molecules such as caspases and BCL-2 [57]. Induction of apoptosis is a promising strategy for combating malignant neoplasms [58]. Previous studies have demonstrated that calcitriol induces apoptosis in breast cancer cells SKBR3 [51], gastric cancer cells SGC and HGC [59], and melanoma cells B16-F10 [27]. Thus, since we did not find alterations in SK-MEL-28 viability, we continued the subsequent analyses focused on A375 cells. Corroborating the data from the literature, we found that calcitriol induced the formation of apoptotic bodies, accompanied by a noticeable decrease in cell counting of A375 as the concentrations of treatment increased (Fig. 3). These results not only confirm the scientific evidence about the pro-apoptotic potential of calcitriol, but also may justify the reduction in the mitochondrial transmembrane potential previously shown in this study.

In advanced stages, CM shows high rates of metastasis [1], spreading to organs such as the lungs, brain, liver, and bones [60]. In this context, metastases involve the dissemination of malignant cells to adjacent tissues and to sites distant from the neoplasm [61]. Hindering the mechanistic processes underlying cancer cell migration may help prevent metastasis [62]. Despite this, our understanding of metastasis remains incomplete, and the development of effective anti-metastatic drugs is urgently needed [63]. In this study, we found that calcitriol inhibited the migration of A375 cells at all concentrations tested (Fig. 4), which have an invasive and metastatic profile. This finding supports a potential role for calcitriol in preventing CM cell migration and metastasis.

As mentioned earlier, we focused on understanding the modulatory effects of calcitriol on purinergic signaling in A375 cells. Assessing ectonucleotidase activity after calcitriol treatment, we did not find alterations in ATP hydrolysis but observed decreased hydrolysis of ADP at 1 nM and 10 nM, with a slight increase at 50 nM. Conversely, the hydrolysis of AMP was reduced in both 10 nM and 50 nM calcitriol concentrations (Fig. 5). It is well-known that levels of ATP, ADP, AMP, and Ado, molecules involved in purinergic signaling, are controlled by the ectonucleotidases’ hydrolytic activity [64], and their modulation may favor an anticancer outcome when Ado generation is inhibited [65, 66]. Therefore, our results demonstrated that calcitriol modulated purinergic signaling by altering ectonucleotidase activity.

The alterations in ectonucleotidase activity we observed were consistent with a purinergic cascade leading to decreased Ado levels. To firmly confirm the effect of calcitriol on Ado generation, we also assessed ADA activity. Ado is a metabolite of AMP [67] and exhibits immunosuppressive effects in the tumor microenvironment (TME) [68]. On the other hand, ADA is the ectoenzyme responsible for the breakdown of Ado into inosine [69]. Consistent with the reduction in AMP hydrolysis, we detected a strong reduction in ADA activity in cells treated with 10 nM and 50 nM calcitriol (Fig. 5D), which is explained by decreased AMP hydrolysis, thereby reducing substrate availability and consequently ADA activity. Thus, our findings corroborated the anticancer effect of calcitriol on A375 cells through modulation of ectonucleotidases, particularly the AMP/Ado axis.

Outside of the cell membrane, the ectonucleotidase CD39 promotes the conversion of ATP into ADP/AMP, while CD73 catalyzes the hydrolysis of AMP into Ado [8]. Thus, together with ADA, CD39 and CD73 play pivotal roles in purinergic signaling in the TME [68]. Overexpressed CD73 has already been demonstrated in different malignant neoplasms, such as pancreatic adenocarcinoma [70], thyroid cancer [71], and cutaneous melanoma [72]. Due to their roles in cancer, CD39 and CD73 have been identified as new targets for cancer therapy [73, 74]. Since it was verified that ectonucleotidase enzymatic activity changed upon treatment of A375 cells, we investigated whether calcitriol also altered their gene expression. Curiously, we found that calcitriol treatment decreased CD39 and CD73 gene expression at the same concentrations at which we observed modulation of ectonucleotidase activity (Fig. 6A-B). Thus, all results corroborate calcitriol’s ability to modulate purinergic signaling, favoring melanoma cell death, and reveal its potential as an adjuvant therapy.

Evidence shows that purinergic signaling can stimulate the release of interleukins that act as effectors of cell death, such as IL-6 [75]. In cancer, studies indicate a dual role for IL-6 in the TME, inducing apoptosis or promoting cancer progression, depending on the pathway by which it is secreted [25, 26]. Findings from the literature indicate that IL-6 is a therapeutic target for abrogating melanoma growth and progression [76–78]. Considering that calcitriol can decrease IL-6 expression via p38 activation [79], we investigated IL-6 expression in A375 cells after treatment. The results showed a decrease in IL-6 expression in cells treated with 10 nM and 50 nM calcitriol (Fig. 6C), indicating that calcitriol reduces IL-6 expression and enhances the anti-melanoma effect.

Similar to IL-6, we found that 10 nM and 50 nM concentrations downregulated NLRP3 gene expression in A375 cells (Fig. 6D). NLRP3 is a component of the inflammasome and is associated with immunity and IL-1β maturation [80, 81]. Previous evidence linked NLRP3 expression to cancer chemoresistance [82]. Recent studies have also shown that NLRP3 is responsible for immune cell infiltration [83] and a bad prognosis in melanoma [84]. Therefore, NLRP3 may be a target in cancer therapy [85]. Thus, based on our results, calcitriol may be effective in targeting NLRP3 and reducing CM progression and chemoresistance.

Given the effects reported in this study, calcitriol appears to be a promising adjuvant therapy for fighting CM. In this context, calcitriol was shown to enhance the anticancer effects of both cisplatin and dacarbazine, widely used chemotherapeutic agents in melanoma treatment [86]. Recently, a study demonstrated that the combination of Cediranib with calcitriol exhibits a superior antiproliferative effect compared to Cediranib alone in melanoma cells [87, 88]. It was also reported in a prospective, non-randomized phase Ib study that patients with metastatic melanoma who received high doses of encapsulated calcitriol (0.2 to 0.5 mcg/kg) in combination with Temozolomide had good tolerability to the treatment, with no significant secondary side effects [89]. Nevertheless, the adjuvant effects of calcitriol are not limited to melanoma pharmacotherapy. It was shown that combining calcitriol with radiotherapy enhanced the inhibition of melanoma cells [90].

In summary, these findings revealed that calcitriol is an active form of vitamin D with promising antineoplastic properties and could be used as an adjuvant in the context of CM. In addition, our results revealed that calcitriol modulates purinergic signaling by downregulating CD39 and CD73 expression and modulating their enzymatic activity. Therefore, based on our results, the nanomolar concentrations used in this study seem practical for in vivo applications but require safety testing. We also recommend that future in vivo studies monitor calcium levels to prevent hypercalcemia and its related adverse effects.

## Study limitations

Given the different metabolic profiles of A375 and SK-MEL-28 cells, including additional cell lines would strengthen the evidence for calcitriol’s effects on melanoma cells, which we recognize as a limitation of our study. Additionally, in our research, we analyzed gene expression for the targets studied but did not measure protein levels.

## Data Availability

The datasets generated during and/or analyzed during the current study are not publicly available but are available from the corresponding author upon reasonable request.
